# Combination therapy with protein kinase inhibitor H89 and Tetrandrine elicits enhanced synergistic antitumor efficacy

**DOI:** 10.1186/s13046-018-0779-2

**Published:** 2018-06-04

**Authors:** Man Yu, Ting Liu, Yicheng Chen, Yafang Li, Wenhua Li

**Affiliations:** Hubei Key Laboratory of Cell Homeostasis, College of Life Sciences, Wuhan University, Wuhan, 430072 People’s Republic of China

**Keywords:** Combination therapy, H89, Tetrandrine, Apoptosis, Autophagy, C-Myc

## Abstract

**Background:**

Tetrandrine, a bisbenzylisoquinoline alkaloid that was isolated from the medicinal plant *Stephania tetrandrine* S. Moore, was recently identified as a novel chemotherapy drug. Tetrandrine exhibited a potential antitumor effect on multiple types of cancer. Notably, an enhanced therapeutic efficacy was identified when tetrandrine was combined with a molecularly targeted agent. H89 is a potent inhibitor of protein kinase A and is an isoquinoline sulfonamide.

**Methods:**

The effects of H89 combined with tetrandrine were investigated in vitro with respect to cell viability, apoptosis and autophagy, and synergy was assessed by calculation of the combination index. The mechanism was examined by western blot, flow cytometry and fluorescence microscopy. This combination was also evaluated in a mouse xenograft model; tumor growth and tumor lysates were analyzed, and a TUNEL assay was performed.

**Results:**

Combined treatment with H89 and tetrandrine exerts a mostly synergistic anti-tumor effect on human cancer cells in vitro and in vivo while sparing normal cells. Mechanistically, the combined therapy significantly induced cancer cell apoptosis and autophagy, which were mediated by ROS regulated PKA and ERK signaling. Moreover, Mcl-1 and c-Myc were shown to play a critical role in H89/tetrandrine combined treatment. Mcl-1 ectopic expression significantly diminished H89/tetrandrine sensitivity and amplified c-Myc sensitized cancer cells in the combined treatment.

**Conclusion:**

Our findings demonstrate that the combination of tetrandrine and H89 exhibits an enhanced therapeutic effect and may become a promising therapeutic strategy for cancer patients. They also indicate a significant clinical application of tetrandrine in the treatment of human cancer. Moreover, the combination of H89/tetrandrine provides new selectively targeted therapeutic strategies for patients with c-Myc amplification.

**Electronic supplementary material:**

The online version of this article (10.1186/s13046-018-0779-2) contains supplementary material, which is available to authorized users.

## Background

Cancer is a multigenic disease caused by the abnormal proliferation and differentiation of cells governed by tumorigenic factors [1]. Chemotherapy is one of the major cancer treatment strategies, and it functions by targeting the biological capabilities of cancer cells, including sustained proliferation, the evasion of programmed cell death and tissue migration [2]. Remarkably, among the FDA approved anticancer drugs, more than 75% originate from natural sources (e.g., Taxol, doxorubicin, or vincristine) and are used in their actual form or with simple modifications from the actual form [3, 4]. Thus, natural products have garnered increased attention in the chemotherapy drug discovery field because they are biologically friendly and have high therapeutic effects [5, 6].

Tetrandrine (Tet), a bisbenzylisoquinoline alkaloid isolated from the medicinal plant *Stephania tetrandrine* S. Moore, has been widely used as an effective agent to treat patients with hypertension, arrhythmia, arthritis, inflammation, and silicosis in traditional Chinese medicine [7]. Of note, tetrandrine has recently been identified as a potential leading compound among anticancer agents with various pharmacological effects, including the regulation of cell viability, migration, invasion, angiogenesis and multidrug resistance of tumors [8, 9]. Our previous studies have indicated that tetrandrine induced apoptosis at a high concentration and induced autophagy at low concentrations [10–12]. Moreover, tetrandrine showed potential anti-tumor activity in leukemia and hepatocellular carcinoma [13, 14].

However, tetrandrine, as a promising chemotherapeutic candidate, was in the preclinical phase [9, 12]. At times, it has been observed that certain phytochemicals are active only when they are in combination with other metabolites of the source material [15]. In addition, as a result of the complexity of cancer with the involvement of multiple signaling pathways, it is difficult for a single compound to combat cancer [16, 17]. Nevertheless, if a compound exhibits a potent anticancer effect, there is a chance for the development of resistance against the compound by tumor cells, thereby making the drug ineffective [18]. Thus, combination therapy may be an available strategy to improve the treatment efficacy [19, 20]. Increasing studies have shown that tetrandrine may induce synergistic activity to enhance cytotoxicity when combined with molecularly targeted drugs, such as sorafenib [21], methylprednisolone [22] and glucocorticoids [23].

H89, a potent protein kinase A (PKA) inhibitor, has the ability to readily cross the cell membrane, with preclinical activity demonstrated in vitro and in vivo [24–26]. H89 attenuates airway inflammation in mouse models of asthma [27]. Of note, recent efforts have focused on its pharmacological activities against cancer. Numerous studies have demonstrated that H89 showed chemotherapy sensitization activity. Reports have documented that H89 enhanced HA22 (Moxetumomab pasudotox) treatment of CD22-positive ALL and mesothelin-expressing solid tumors [28]. H89 has also been shown to dramatically synergize with oncolytic virus M1 to improve tumor regression and trigger apoptosis in aggressive cancer cells when combined with glyceryl trinitrate (GTN) [29, 30].

In this work, we discovered that H89 and tetrandrine showed synergistic anti-tumor effects on various cancer cells in vitro and in vivo, and we investigated the underlying mechanisms of their anti-tumor activities. In addition, we determined that c-Myc amplified cells are more sensitive to H89/tetrandrine combined treatment, which may represent a novel, selective therapeutic strategy for cancer patients.

## Methods

### Cell lines and cell culture

The human breast cancer cell lines (MDA-MB-231, MDA-MB-468, and MCF-7) were purchased from ATCC (Manassas, VA, USA). The human hepatoma cell lines (Hep3B and Huh7) and the normal cell lines (L02, HBL-100, and HEK293T) were purchased from CCTCC (Wuhan, China). The cell line HCCLM9 was purchased from the Liver Cancer Institute (Fudan University, China). These various cell lines were cultured in DMEM. The human renal carcinoma cell lines (769-P, ACHN, and 786-O) and the human lung cancer cell line (A549) were purchased from ATCC and maintained in 1640 RPMI. The human colon cancer cell lines (LOVO and HCT116) were purchased from ATCC and cultured in McCoy’s 5A. All cells were cultured in media supplemented with 10% fetal bovine serum (FBS, HyClone), penicillin 100 U·mL^− 1^ and 100 μg·ml^− 1^ streptomycin and were incubated at 37 °C in a humidified atmosphere with 5% CO_2_.

### Reagents

The reagents used in this study are listed as follows: H89 2HCl (10 mM, dissolved in DMSO, S1582, Selleck, Houston, TX), forskolin (FSK) (10 mM, dissolved in DMSO, S2449, Selleck, Houston, TX), PKI (14–22) amide myristoylated (PKI) (0.5 mg, dissolved in DMSO, Sc471154, Santa Cruz, CA), Tetrandrine (10 mM, dissolved in DMSO, CAS:518–34-3, Shanghai Ronghe Medical, Shanghai, China), Caspase inhibitor z-VAD-fmk (10 mM, dissolved in DMSO, S7023, Selleck, Houston, TX), Bafilomycin A1 (dissolved in DMSO, S1413, Selleck, Houston, TX), Chloroquine (CQ) (C6628, Sigma-Aldrich, USA), PD98059 (Beverly, MA, USA), N-acetyl-L-cysteine (NAC) (dissolved in ddH_2_O, A0150000, Sigma-Aldrich, USA), 3-Methyladenine (3-MA) (dissolved in ddH_2_O, M9281-100MG, Sigma-Aldrich, USA), 2′,7′-dichlorodihydrofluorescein diacetate (DCFH-DA) (Invitrogen Carlsbad, CA), and an FITC Annexin V Apoptosis Detection Kit (556,547, BD Pharmingen). All reagents were formulated as recommended by their suppliers.

### Cell viability assay

To measure viability following H89 and/or tetrandrine treatment, cells were seeded on 96-well plates at a density of 4 × 10^3^ cells per well. Cells were allowed to attach overnight in complete media and were subsequently treated with the indicated concentrations of tetrandrine and/or H89 for an additional 72 h. Control cells received DMSO (< 0.1%) that contained medium. The cell viability was determined using the trypan blue dye exclusion assay according to established protocols.

### Drug combination analysis

A drug combination analysis was performed using the method described by Chou and Talalay [31]. Briefly, cells were plated in 96-well plates and treated with 1–5 μM tetrandrine and 1–10 μM H89 alone or in combination for 72 h; the cell viability was subsequently assessed. Multiple drug dose-effect calculations and the combination index plots were generated using Calcusyn 2.1 software (Biosoft, Cambridge, UK). CI values < 1, = 1 and > 1 indicate synergism, additive and antagonism between two drugs, respectively.

### Apoptosis assay

Apoptosis was determined using an Annexin V-FITC/PI apoptosis detection kit (BD Biosciences, San Jose, CA, USA) according to the manufacturer’s instructions. Briefly, the untreated and treated cells were washed with PBS and gently suspended in Annexin V binding buffer, followed by incubation with Annexin V-FITC and PI at room temperature for 15 min. Finally, the fluorescent intensities were determined by a flow cytometer (Beckman Coulter, Indianapolis, CA, USA), and the data were analyzed using FlowJo software (Tree Star Inc., San Carlos, CA, USA).

### Colony formation assay

Cell were seeded at 2500 cells per well in 6-well plates and treated with H89, tetrandrine or the combination. Cells were washed with fresh medium after 24 h, allowed to grow for 8 days under drug-free conditions, and stained with crystal violet (Sigma-Aldrich, USA). Colonies with more than 50 cells were counted.

### Western blot and antibodies

After various treatments, both floating and adherent cells were harvested and subsequently washed with cold PBS. The cells were then lysed with 1% SDS on ice. The cell lysates were subsequently heated at 95 °C for 20 min and centrifuged at 12,000×*g* for 10 min, and the supernatant was collected. For the tumor tissue, the samples were homogenized and sonicated in RIPA buffer (Beyotime, Nantong, China) in the presence of protease inhibitor cocktail on ice. The tissue lysates were subsequently centrifuged at 12,000×*g* for 15 min at 4 °C, and the supernatant was collected for Western blotting analysis. Protein was quantified using a BCA Protein Assay Kit (Thermo Scientific, MA, USA). The proteins were separated by 8–12% SDS-PAGE and transferred to PVDF membranes (Millipore, Billerica, MA, USA). The blots were blocked for 2 h at room temperature with freshly prepared 5% nonfat milk (Bio-Rad, USA) in TBST and were subsequently incubated with specific primary antibodies overnight at 4 °C. The membranes were then washed with TBST and incubated with HRP conjugated secondary antibodies for 1 h at room temperature. After washing with TBST, the immunoblots were visualized by Immobilon™ Western HRP Substrate peroxide (Millipore, Billerica, MA, USA). The following antibodies were employed: anti-PARP (# 9542), anti-Caspase-3 (# 9662), anti-Caspase-9 (# 9502), anti-Bcl-2 (# 2872), anti-Bcl-xL (# 2762), anti-Mcl-1 (# 4572), anti-Bim (# 2819), anti-Bid (# 2002), anti-cytochrome c (# 4272), anti-p-ERK1/2 (Thr202/Tyr204, # 9101), anti-T-ERK (# 9102), p-MEK (# 9121S), anti-p-CREB1-s133 (# 9198), and anti-CREB1 (# 9197), which were obtained from Cell Signaling Technology. Antibodies for LC3B (# L7543) and anti-β-actin (# A2228) were obtained from Sigma-Aldrich. Antibody for c-Myc (9E10) (sc-40) was obtained from Santa Cruz Biotechnology.

### Determination of intracellular ROS

The intracellular ROS levels were detected by a flow cytometer utilizing Dichloro-dihydro-fluorescein diacetate (DCFH-DA). Briefly, 7 × 10^4^ cells were plated on 12-well plates, allowed to attach overnight and then treated with the indicated concentrations of tetrandrine and/or H89 for the indicated times. NAC pretreatment, where indicated, was conducted for 1 h. Cells were stained with DCFH-DA (1 μM) in serum-free media at 37 °C for 30 min in the dark. DCF fluorescence (produced in the presence of ROS) was analyzed using flow cytometry. For the detection of the mitochondrial membrane potential, the cell pretreatments were performed as described for the ROS detection protocol. A 5 μL solution of 0.1 mg·mL^− 1^ Rho123 (Sigma-Aldrich, USA) was added, and the cells were incubated for 30 min at 37 °C. The fluorescence was measured via flow cytometry.

### Tandem mRFP-GFP-LC3 reporter assay

Cells were transfected with the mRFP-GFP-LC3 plasmid (Addgene, # 21074) and were subsequently treated with DMSO or H89/tetrandrine. Autophagy flux was assessed by counting cells that were mRFP^+^GFP^+^LC3 (yellow puncta), which represents autophagosomes, and mRFP^+^GFP^−^LC3 (red puncta), which represents autolysosomes.

### Lentiviral transduction

pLKO.1 plasmids that contained shRNA sequences targeting c-Myc (shc-Myc#1: target sequence: CCTGAGACAGATCAGCAACAA; shc-Myc#2: target sequence CAGTTGAAACACAAACTTGAA) were established. The empty vector was used as a negative control. PHAGE-puro-c-Myc and the control plasmids were kindly provided by Dr. Youjun Li (Wuhan University). A PHAGE-puro-Mcl-1 plasmid was constructed. To generate virus, 293 T cells were seeded in each 10 cm dish. After 24 h, the control and shRNA constructs that targeted c-Myc (12 μg each), packaging plasmid (psPAX2; 6 μg) and envelope plasmid (pMD2.G; 6 μg) were diluted with 0.5 mL Opti-MEM medium (Gibco, Invitrogen, Carlsbad, CA, USA) and mixed with transfection reagent FuGENE™ HD (Roche, USA). Twelve hours after transfection, the culture medium was changed to fresh culture medium; after 48 h, the virus-containing supernatants were collected and used to infect cells in the presence of 8 μg·mL^− 1^ polybrene (Sigma-Aldrich, USA). The cells were subsequently selected in the presence of puromycin (5 μg·mL^− 1^; Sigma-Aldrich, USA) to establish stable clones.

### RT-PCR

RNA was isolated from cultured cells using an OMEGA-RNA Miniprep kit, and RNA was reverse-transcribed into cDNA molecules using a cDNA synthesis kit (Roche Applied Science, USA). The numbers of Mcl-1 and β-actin molecules were monitored in real time on a 7500 Fast Real-Time PCR System (Applied Biosystems) by measuring the fluorescence increases of SYBR Green (Roche Applied Science, USA). The primer sequences for PCR were as follows: Mcl-1 forward 5’-CCAAGAAAGCTGCATCGAACCAT-3′ and Mcl-1 reverse 5’-CAGCACATTCCTGATGCCACCT-3′; β-actin forward 5’-GGCATGGGTCAGAAGGATT-3′ and β-actin reverse 5’-AGGATGCCTCTCTTGCTCTG-3′. To determine the relative abundance of Mcl-1 in relation to β-actin, the Δ-ΔCT (cycle threshold) method was utilized.

### Tumor xenograft

Animals were handled according to the Guidelines of the China Animal Welfare Legislation, as provided by the Committee on Ethics in the Care and Use of Laboratory Animals of Wuhan University. The experimental protocols were approved by the Experimental Animal Centre of Wuhan University. Female nude nu/nu BALB/c mice (14–16 g; 4–5 weeks of age) were purchased from Hunan SJA Laboratory Animal Co., Ltd. (Changsha, China). Animals were housed at a constant room temperature with a 12/12 h light/dark cycle and were fed a standard rodent diet.

MDA-MB-231 cells (5 × 10^6^), MDA-MB-231 control or Mcl-1 cells (5 × 10^6^), or MDA-MB-231 shCtrl or shc-Myc cells (5 × 10^6^) were subcutaneously implanted into the right flank of each mouse in 0.2 mL PBS. Once the tumor volume of MDA-MB-231 cells reached 50–100 mm^3^, the tumor-bearing mice were randomly separated into four groups (*n* = 6) and treated via gavage of 25 mg·kg^− 1^tetrandrine with 0.5% sodium carboxyl methylcellulose, i.p. injection of 10 mg·kg^− 1^ H89 (in a solution of PBS with 1% DMSO+ 30% polyethylene glycol+ 1%Tween 80) or a combination of H89 and tetrandrine (10 mg·kg^− 1^ H89 and 25 mg·kg^− 1^ tetrandrine) every other day for 28 days. The control group received the same vehicle. For the MDA-MB-231 control and Mcl-1 cells, tumor-bearing mice were randomized into two test groups (*n* = 6) and were administered vehicle (0.5% carboxymethylcellulose sodium) or H89 (10 mg·kg^− 1^) and tetrandrine (25 mg·kg^− 1^) every other day for 36 days. For the MDA-MB-231 shCtrl and shc-Myc cells, tumor-bearing mice were randomized into two test groups (*n* = 6) and were administered vehicle (0.5% carboxymethylcellulose sodium) or H89 (10 mg·kg^− 1^) and tetrandrine (25 mg·kg^− 1^) every other day for 30 days. The tumor volumes were determined by measuring the length (l) and width (w) and calculating the volume (V = 0.5 × l × w^2^) every other day. At the end of the experiments, the mice were euthanized after being anesthetized with an i.p. injection with pentobarbital (50 mg·kg^− 1^); their tumors were isolated by dissection, weighed and used for in vitro experiments. Samples were prepared for histology and protein assays. All studies involving animals are reported in accordance with the ARRIVE guidelines for reporting experiments involving animals [32].

### Malondialdehyde (MDA) assay

Tumor samples from mice were homogenized and sonicated. Tissue lysates were subsequently centrifuged at 12000×g for 10 min at 4 °C to collect the supernatant. The total protein content was determined using the Bradford assay. The MDA levels were measured by the Lipid Peroxidation MDA assay kit (Beyotime Institute of Biotechnology).

### Statistical analysis

All experiments were randomized and repeated at least three times. Data analysis was performed using Microsoft Excel and GraphPad Prism Software version 5.0 (GraphPad Software, La Jolla, CA, USA). All data are expressed as the mean ± SD. Student’s two-tailed t-tests were performed to calculate *P* values unless otherwise specified. *P* < 0.05 was considered statistically significant.

## Results

### Combination treatment with H89 and tetrandrine yields synergistic anti-tumor effects in multiple human cancer cells

**Fig. 1:**
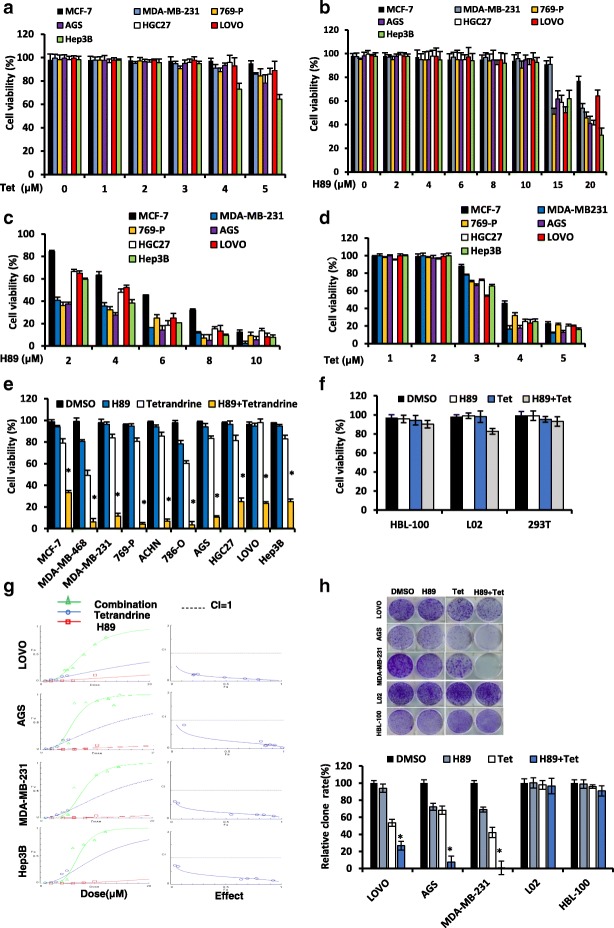
Combination treatment with H89 and tetrandrine yields a synergistic antitumor effect across different types of cancer cells. **a** Cell viabilities were determined by a trypan blue dye exclusion assay in cancer cells treated with increasing doses of tetrandrine (0–5 μM) or (**b**) H89 (0–20 μM) alone for 72 h. **c** Cancer cells were exposed to the designated concentrations of H89 in combination with tetrandrine (4 μM) for 72 h. Cell viabilities were determined as previously described. **d** Cells were exposed to the designated concentrations of tetrandrine in combination with H89 (6 μM) for 72 h, following which the cell viabilities were determined. **e** Cell viabilities were determined in cells incubated with H89 (6 μM) and tetrandrine (4 μM) alone or in combination for 72 h. **f** HBL-100, L02 and HEK293T cells were treated with H89 (6 μM) and tetrandrine (4 μM) alone or in combination for 72 h. Cells viabilities were determined. **g** The combination index (CI) values for LOVO, MDA-MB-231, AGS, and Hep3B cells were constructed by CalcuSyn 2.1 software. **h** Cells were treated with H89 and tetrandrine alone or in combination for 24 h. The attached cells were stained with crystal violet after 8 days. The number of colonies was quantified. All representative images are from three independent experiments. Data are reported as the mean ± SD and were analyzed by Student’s t-test; all data represent at least *n* = 3 independent experiments, *P < 0.05 compared with DMSO control

To examine whether a cooperative effect exists between H89 and tetrandrine in tumor chemotherapy, we treated cancer cells with a series of concentrations of H89 and tetrandrine alone or in combination. Dose-response studies indicated that 0–5 μM tetrandrine or 0–10 μM H89 were only minimally toxic by themselves (Fig. 1, a and b). However, 4 μM tetrandrine substantially increased the cell death of H89 at 6–10 μM (Fig. 1c). Analogously, the lethality of the marginally toxic tetrandrine concentrations (3–5 μM) was significantly increased by co-exposure to 6 μM H89 (Fig. 1d). In addition, we determined that 4 μM tetrandrine plus 6 μM H89 had a clear effect on most human cancer cells (Fig. 1e). In contrast, normal cells, such as HBL-100, L02 and HEK-293 T, were substantially less sensitive to H89/tetrandrine, which suggests that the H89/tetrandrine combined treatment is relatively selective towards cancer cells (Fig. 1f). The use of the Chou-Talalay method to calculate the H89/tetrandrine interaction showed combination indexes < 1, which indicated their synergistic effects under a number of the treatment conditions (Fig. 1g). Furthermore, the analysis of long-term cell survival via colony formation assay indicated that the combination of H89 and tetrandrine suppressed colony formation significantly more than tetrandrine or H89 alone in cancer cells, whereas the effect on normal cells was not significant (Fig. 1h). These data suggest that a combination treatment of H89 and tetrandrine has a significant synergistic anti-tumor effect on cancerous cells.

### H89 in combination with tetrandrine synergistically induced concomitant apoptosis and autophagy

**Fig. 2:**
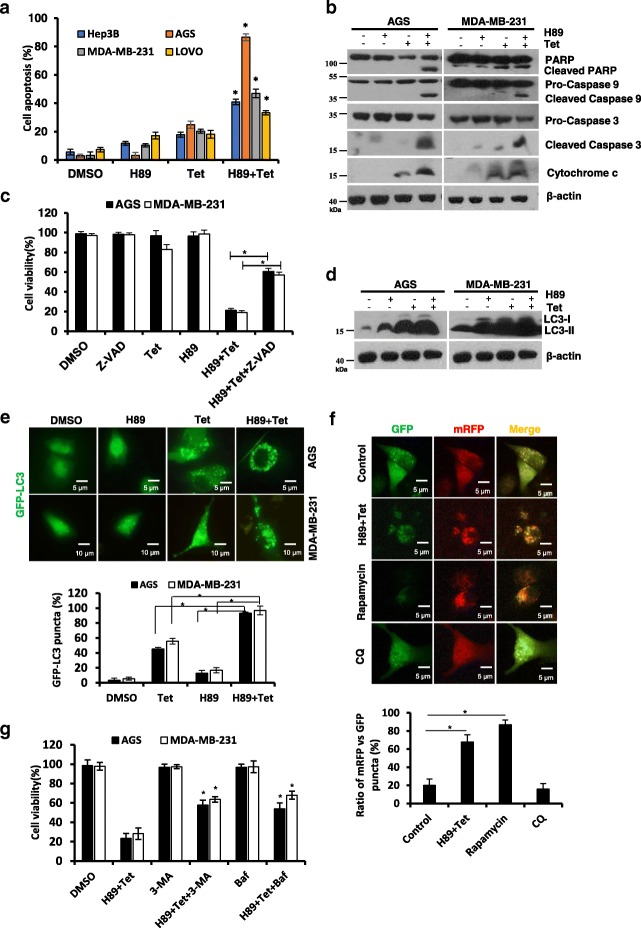
H89 in combination with tetrandrine synergistically induced concomitant apoptosis and autophagy. **a** FACS analysis of apoptosis following treatment with H89 (6 μM) and tetrandrine (4 μM) alone or combination on Hep3B, AGS, MDA-MB-231, and LOVO for 48 h. **b** Western blot analysis of PARP, caspase 9, caspase 3 and cytochrome c in the cells after treatment with H89 and/or tetrandrine for 48 h. **c** The cells were pretreated with z-VAD-fmk (50 μM) for 1 h, followed by treatment with H89 and/or tetrandrine for 72 h; cell viabilities were subsequently evaluated. Z-VAD, z-VAD-fmk. **d** Western blot analysis of the level of the autophagy-related protein LC3 in the cells after treatment with H89 and/or tetrandrine for 24 h. **e** Cancer cells were transiently transfected with GFP-LC3 plasmid and subsequently treated as in panel (**d**); the percentage of cells with GFP-LC3 puncta was used to quantify the percentage of autophagic cells, and 150–160 cells per condition were counted. Representative images are shown to indicate the cellular localization patterns of the GFP-LC3 fusion protein (× 40 magnification). **f** Fluorescence microscopy analysis of AGS cells expressing tandem mRFP-GFP-LC3 reporter treated with H89/tetrandrine, rapamycin (500 nM) and chloroquine (10 μM) for 36 h; the ratio of mRFP vs GFP puncta was used to quantify the autophagy flux. Scale bars, 5 μm. **g** Cell viability was determined in cells pretreated with 3-MA (2 mM) or bafilomycin A1 (100 nM) for 1 h following H89/tetrandrine combination treatment for 48 h. Baf, Bafilomycin A1. Data are reported as the mean ± SD and were analyzed by Student’s t-test; all data represent at least *n* = 3 independent experiments; *P < 0.05. All images are representative of three independent experiments

To determine whether the reduction in cell survival induced by H89/tetrandrine was associated with cell apoptosis, cancer cells were treated with H89 and tetrandrine alone or in combination for 48 h and were subsequently analyzed via flow cytometry with Annexin V/PI. As shown in Fig. 2a and Additional file 1: Figure S1A, H89/tetrandrine combination treatment increased apoptotic cell death compared with each agent alone. Consistently, apoptosis was also evident from Western blots, which indicated that the cleavages of PARP, caspase-3, caspase-9 and cytochrome c were substantially increased in the combination treatment (Fig. 2b). To ascertain whether H89/tetrandrine induced cell apoptosis was dependent on caspase activation, we pre-treated cells with z-VAD-fmk for 1 h. The results showed that z-VAD-fmk partially rescued H89/tetrandrine induced cell death (Fig. 2c). Our previous studies have shown that tetrandrine is a potent autophagy agonist. We subsequently examined whether the H89/tetrandrine combination synergistically induced autophagy. Western blot and GFP-LC3 fluorescence assays showed that the H89/tetrandrine combination treatment synergistically increased autophagy (Fig. 2d and e, and Additional file 1: Figure S1B). In addition, we used an mRFP-EGFP-LC3 tandem-tagged fluorescent protein (ptf-LC3) to determine the dynamic process of autophagic flux [33, 34]. The results suggested that the combination of H89/tetrandrine promotes functional autophagy in cancer cells (Fig. 2f). Next, to investigate the role of autophagy in cell death, we pre-treated cells with 3-MA or bafilomycin A1 for 1 h. As shown in Fig. 2g, the autophagy inhibitor also partially inhibited H89/tetrandrine induced cell death. Collectively, these data indicate that apoptosis and autophagy act simultaneously in H89/tetrandrine induced cancer cell death.

### H89/tetrandrine induced cell death is associated with the generation of ROS

**Fig. 3:**
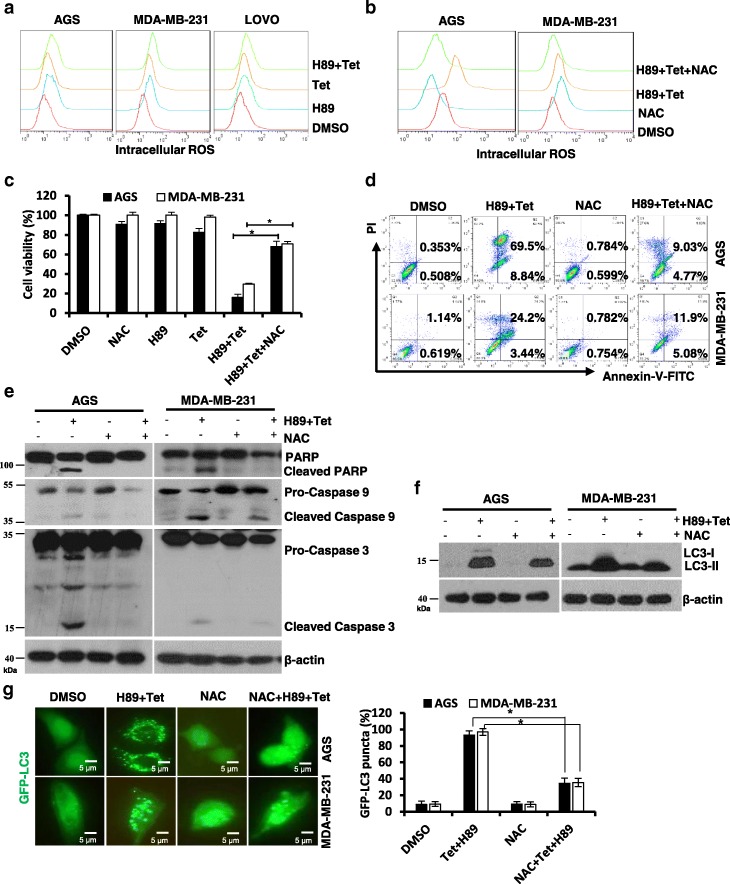
H89/tetrandrine induced cell death is associated with ROS generation. **a** The intracellular ROS were detected by flow cytometry after the cells had been treated with 6 μM H89 and 4 μM tetrandrine alone or in combination for 24 h. **b** The cells were pretreated with 15 mM NAC for 1 h, followed by treatment with DMSO or H89/tetrandrine. The intracellular ROS were measured by flow cytometry after 24 h of treatment. **c** Cell viability was determined after 72 h of treatment. **d** Apoptosis was measured by flow cytometry, and (**e**) PARP, caspase 3, and caspase 9 levels were analyzed by Western blot after 48 h of treatment. **f** After 24 h of treatment, LC3 expression was measured by Western blot and (**g**) GFP-LC3 was observed using a fluorescence microscope. Scale bars, 5 μm. Data are reported as the mean ± SD and were analyzed by Student’s t-test; all data represent at least *n* = 3 independent experiments; *P < 0.05 compared with DMSO control. All images are representative of three independent experiments

Increasing evidence suggests that chemotherapeutic agents activate intracellular reactive oxygen species (ROS) accumulation and subsequently induce cell death [35, 36]. We assessed the intracellular ROS levels via flow cytometry with DCFH-DA, which specifically detects peroxides. As shown in Fig. 3a, the H89/tetrandrine combination treatment substantially increased intracellular ROS compared with either agent alone in cancer cells. Pretreatment of cells with the ROS scavenger NAC not only strikingly abrogated H89/tetrandrine-induced ROS production (Fig. 3b) but also significantly rescued them from cell death induced by combination treatment (Fig. 3c). Moreover, NAC rescued cells from apoptosis (Fig. 3d) and blocked both PARP cleavage and caspase 3/caspase 9 activation in AGS and MDA-MB-231 cells (Fig. 3e). In addition, pretreatment with NAC also significantly decreased the intracellular LC3-II expression and GFP-LC3 fluorescent punctate dots induced by H89/tetrandrine (Fig. 3f and g). Therefore, these results clearly demonstrated that the H89/tetrandrine combination treatment-induced apoptosis and autophagy were initiated by ROS production.

### PKA and ERK signaling are involved in H89/tetrandrine induced cell death

**Fig. 4:**
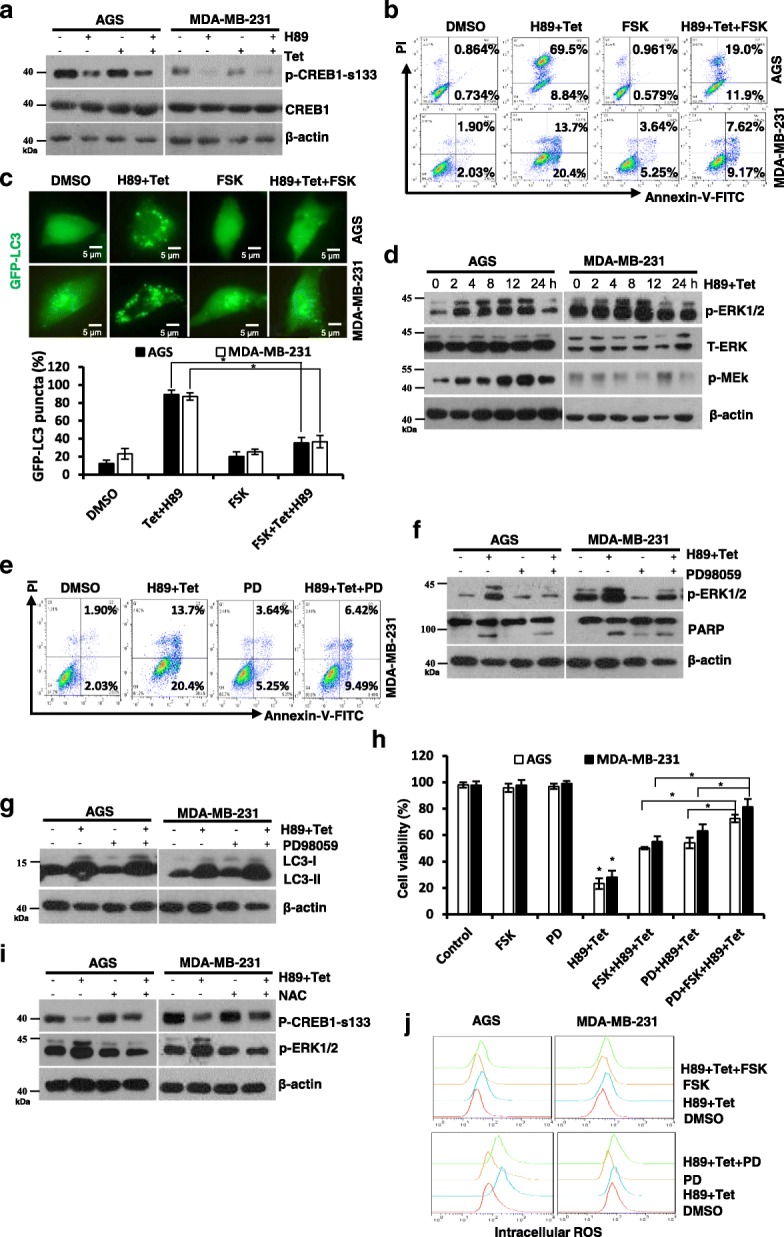
PKA and ERK signaling are involved in H89/tetrandrine induced cell death. **a** Western blot of p-CREB1-s133 and CREB1 in AGS and MDA-MB-231 cells exposed to H89 and/or tetrandrine for 24 h. **b** The cells were pre-treated with FSK (10 μM) for 1 h, followed by treatment with DMSO or H89/tetrandrine, and apoptosis was measured by flow cytometry after 36 h of treatment. **c** GFP-LC3 punctate was determined using a fluorescence microscope after 24 h of treatment. Scale bars, 5 μm. **d** Western blot of p-ERK1/2, T-ERK and p-MEK in cells incubated with H89/tetrandrine for 0, 2, 4, 8, 12 & 24 h. **e** MDA-MB-231 was pre-treated with 20 μM PD98059 for 1 h prior to exposure to H89/tetrandrine. Apoptosis was detected by flow cytometry after 36 h, and (**f**) p-ERK and PARP levels were analyzed by Western blot at 8 h and 36 h, respectively. **g** After 24 h of treatment, LC3 expression was measured by Western blot. **h** Cells were pre-treated with PD98059 and FSK alone or in combination for 1 h prior to exposure to H89/tetrandrine for 72 h. Cell viability was determined by trypan blue staining. PD, PD98059. **i** Analysis of p-CREB1-s133 and p-ERK levels in the cells pretreated with NAC for 1 h prior to exposure to H89/tetrandrine for 8 h. **j** The cells were pre-treated with FSK or PD98059 for 1 h followed by H89/tetrandrine combination treatment for 24 h; intracellular ROS were subsequently detected by flow cytometry. Data are reported as the mean ± SD and were analyzed by Student’s t-test; all data represent at least *n* = 3 independent experiments; *P < 0.05 compared with DMSO control. All images are representative of three independent experiments

H89 is a strong inhibitor of cyclic AMP-dependent protein kinase A (PKA) [37]. To determine whether PKA activity is related to synergistic antitumor activity, we initially detected the levels of phosphorylated cAMP-dependent response element (CREB ser133). As shown in Fig. 4a, H89 alone or combined with tetrandrine significantly decreased the phosphorylation of the transcription factor CREB in cancer cells. In contrast, forskolin (FSK), an established activator of adenylyl cyclase, significantly reduced the proportion of cells undergoing apoptosis and autophagy (Fig. 4b and c, and Additional file 1: Figure S2, A and B). Our previous studies have shown that tetrandrine activates intracellular ERK [38]. We subsequently examined the role of MEK/ERK signaling in H89/tetrandrine combination treatment. As shown in Fig. 4d, H89/tetrandrine transiently activated MEK/ERK1/2 signaling, and these effects mainly originated from tetrandrine (Additional file 1: Figure S2C). Inhibition of ERK1/2 with PD98059 could partially rescue H89/tetrandrine induced cell apoptosis (Fig. 4e and f), whereas it had no effect on autophagy (Fig. 4g). However, concomitant pretreatment of cells with FSK and PD98059 almost completely restored cell viability, which implies that PKA and ERK signaling are simultaneously involved in H89/tetrandrine induced cell death (Fig. 4h). Moreover, NAC could mitigate H89/tetrandrine regulated PKA and ERK activities, whereas FSK and PD98059 had no effect on H89/tetrandrine increased ROS (Fig. 4i and j), which indicates that PKA and ERK signaling are involved in H89/tetrandrine induced cell death and are initiated by ROS. Therefore, we demonstrated that the inhibition of PKA and activation of ERK activity play a role in H89/tetrandrine induced cell death.

### Mcl-1 plays a critical role in H89/tetrandrine induced cell death

**Fig. 5:**
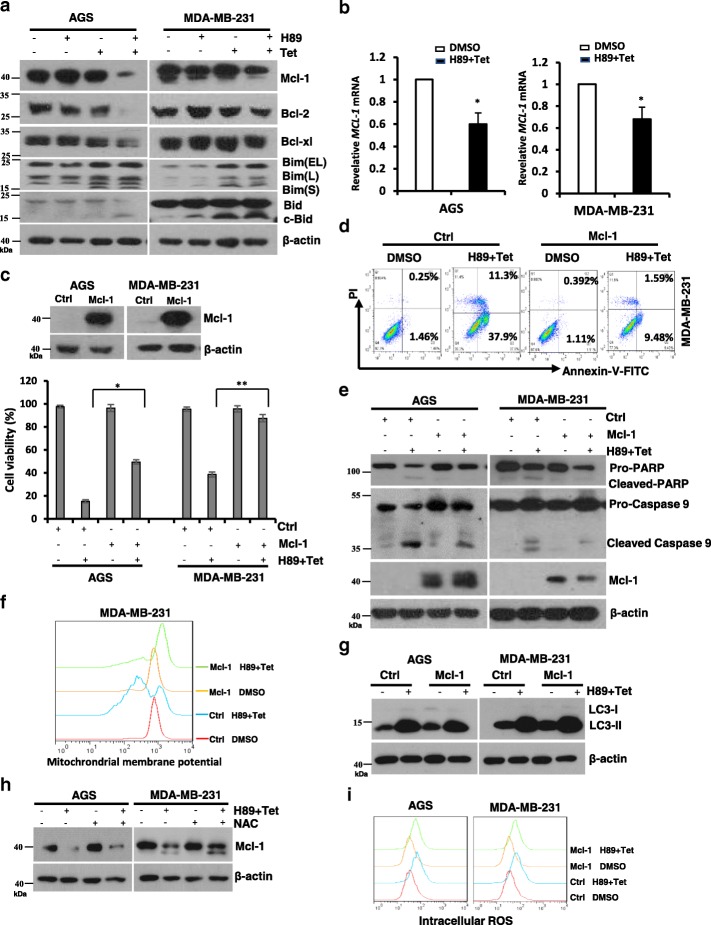
Mcl-1 plays a critical role in H89/tetrandrine induced cell death. **a** Western blot analysis of Bcl-2 family members after treatment for 48 h. **b** AGS and MDA-MB-231 cells were treated with DMSO or H89/tetrandrine for 24 h; the relative Mcl-1 mRNA expression was determined by qPCR, and β-actin was used as a reference. **c** The cells stably transfected with Mcl-1 were treated with DMSO or H89/tetrandrine for 72 h and subjected to viability assays. **d** MDA-MB-231 Mcl-1 overexpression and control cells were treated with DMSO or H89/tetrandrine for 48 h. Apoptosis was evaluated by flow cytometry. **e** Western blot analysis of PARP, caspase 9 and Mcl-1 in the cells after treatment with DMSO or H89/tetrandrine for 48 h. **f** The mitochondrial membrane potential was measured by flow cytometry with Rho123 in the cells after treatment with DMSO or H89/tetrandrine for 48 h. **g** LC3 was determined in the cells after treatment with DMSO or H89/tetrandrine for 24 h. **h** AGS and MDA-MB-231 cells were pre-treated with NAC for 1 h followed by H89/tetrandrine for 36 h; Mcl-1 levels were subsequently determined by Western blot. **i** Cells with control or Mcl-1 overexpression were treated with DMSO or H89/tetrandrine for 24 h; intracellular ROS levels were subsequently measured. Data are reported as the mean ± SD and were analyzed by Student’s t-test; all data represent at least *n* = 3 independent experiments; *P < 0.05, **P < 0.01 compared with DMSO control. All images are representative of three independent experiments

Bcl-2-family proteins play a role in chemotherapy induced cell death, including apoptosis and autophagy [39]. We first detected the Bcl-2, Bcl-xl and Mcl-1 expression levels by Western blotting after AGS and MDA-MB-231 cells underwent treatment with H89 and tetrandrine alone or in combination for 48 h. As shown in Fig. 5a, the expression of Mcl-1 was significantly decreased after H89/tetrandrine treatment in both AGS and MDA-MB-231 cells. Consistent with its protein level, the Mcl-1 mRNA expression was downregulated by H89/tetrandrine (Fig. 5b). We subsequently assessed the role of Mcl-1 in H89/tetrandrine mediated cell death. AGS and MDA-MB-231 were established stably expressing a control or Mcl-1 and were subsequently treated with H89/tetrandrine. In the presence of H89/tetrandrine, overexpression of Mcl-1 diminished cell sensitivity to the combined treatment (Fig. 5c) and attenuated cell apoptosis (Fig. 5d and e); however, it had no effect on autophagy (Fig. 5g). As an anti-apoptotic Bcl-2 family member, Mcl-1 appeared to inhibit apoptosis by preventing mitochondrial dysfunction [40]. We determined that overexpression of Mcl-1 significantly rescued the H89/tetrandrine-induced mitochondrial transmembrane potential drop (Fig. 5f). In addition, NAC could partially impede the H89/tetrandrine triggered Mcl-1 decrease (Fig. 5h); however, Mcl-1 overexpression had no significant effect on the ROS increases (Fig. 5i), which demonstrates that the induction of intracellular ROS occurs upstream of the inhibition of Mcl-1 activity. Therefore, we concluded that the activity of the anti-apoptotic protein Mcl-1 considerably contributes to H89/tetrandrine induced cell death.

### C-Myc sensitizes cancer cells to H89/tetrandrine combination treatment

**Fig. 6:**
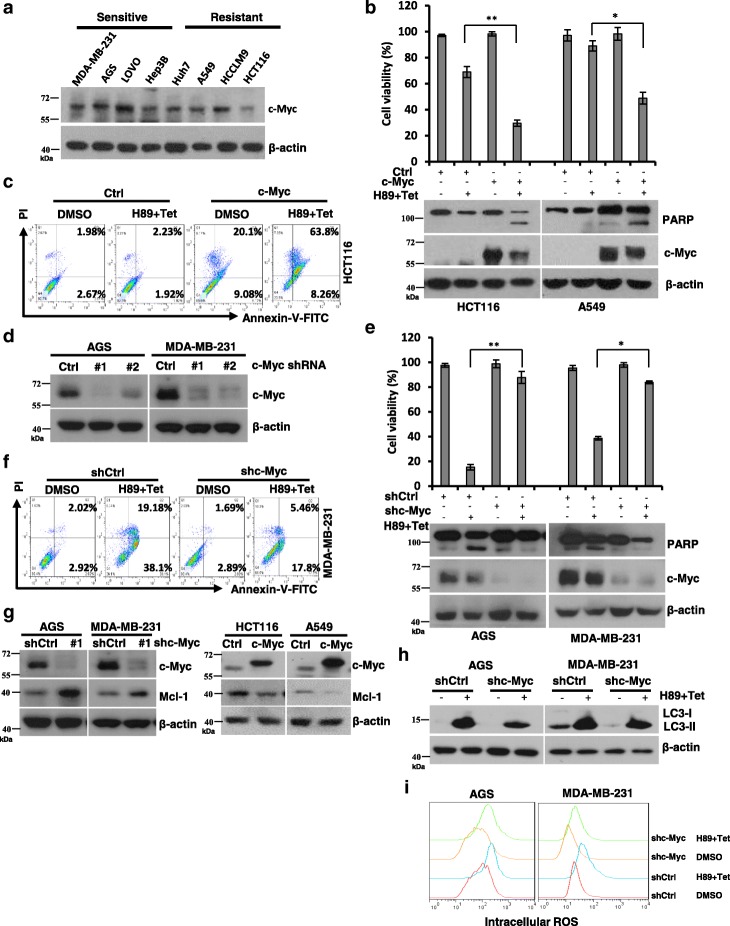
c-Myc sensitizes cancer cells to H89/tetrandrine combination treatment. **a** Western blot of c-Myc in H89/tetrandrine sensitive cells (MDA-MB-231, AGS, LOVO and Hep3B) and resistant cells (Huh7, A549, HCCLM9 and HCT116). **b** HCT116 and A549 cells engineered to overexpress c-Myc and exposed to H89/tetrandrine combined treatment. Cell viabilities and PARP activation were determined at 72 h and 48 h, respectively. **c** Apoptosis was detected by flow cytometry in HCT116 c-Myc overexpressing cells after H89/tetrandrine combination treatment for 48 h. **d** AGS and MDA-MB-231 cells were stably transduced with lentiviral vectors that expressed c-Myc shRNAs (#1 or #2) and negative control vector PLKO.1 (shCtrl); c-Myc protein levels were subsequently detected to confirm the knockdown efficiency. **e** shRNA mediates c-Myc knockdown in AGS and MDA-MB-231 cells undergoing H89/tetrandrine combination treatment. Cell viabilities and PARP were determined as previously described. **f** MDA-MB-231 knockdown of c-Myc following treatment with H89/tetrandrine for 48 h. Apoptosis was detected by Annexin V/PI staining. **g** c-Myc and Mcl-1 levels were determined by Western blot in AGS and MDA-MB-231 cells after knockdown of c-Myc and HCT116, A549 cells overexpressing c-Myc. **h** Western blot of LC3 in AGS and MDA-MB-231 c-Myc knockdown cells following H89/tetrandrine combination treatment for 24 h. **i** AGS and MDA-MB-231 cells were transduced with c-Myc shRNA#1 or shCtrl. Intracellular ROS were determined by flow cytometry after H89/tetrandrine treatment for 24 h. Data are reported as the mean ± SD and were analyzed by Student’s t-test; all data represent at least *n* = 3 independent experiments; *P < 0.05, **P < 0.01. All images are representative of at least three independent experiments

Deregulated and enhanced expression of c-Myc contributes to the genesis of a substantial fraction of human tumors [41]. Paradoxically, high expression levels of c-Myc not only promote transformation but also sensitize cells against a broad range of pro-apoptotic stimuli, such as growth factor deprivation, hypoxia, ionizing radiation, or exposure to chemotherapy [42–44]. Consistently, in this study, we determined that H89/tetrandrine sensitive cells showed higher levels of c-Myc expression than resistant cells (Fig. 6a, Fig. 1e, and Additional file 1: Figure S3A). To further verify this observation, we ectopically expressed c-Myc in HCT116 and A549 cells, which express low levels of c-Myc and are resistant to H89/tetrandrine treatment. Interestingly, the overexpression of c-Myc enhanced the effects of combination treatment induced cell death and apoptosis (Fig. 6b and c). In contrast, the knockdown of c-Myc in AGS and MDA-MB-231, which express high levels of c-Myc, decreased the sensitivity of cells to H89/tetrandrine (Fig. 6d-f). Importantly, we determined that c-Myc significantly regulated Mcl-1 expression (Fig. 6g). Furthermore, we determined that knockdown of c-Myc in AGS and MDA-MB-231 cells, cell autophagy, and intracellular ROS generation triggered by H89/tetrandrine combination treatment were suppressed (Fig. 6h and i). In contrast, ectopically expressed c-Myc in HCT116 and A549 cells significantly elevated intracellular ROS (Additional file 1: Figure S3B). Together, these data indicate that c-Myc amplification downregulates Mcl-1 expression and increases intracellular ROS, which contributes to H89/tetrandrine sensitivity.

### Combination of H89 and tetrandrine causes tumor regression in xenograft models

**Fig. 7:**
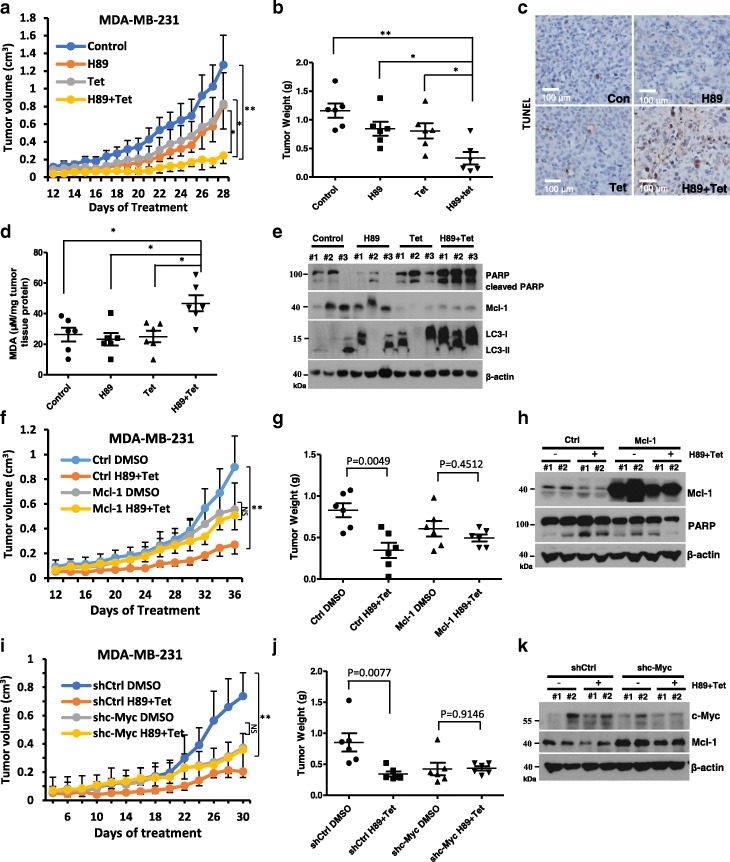
Combination treatment with H89 and tetrandrine leads to a regression of xenograft tumors. **a** MDA-MB-231 cells were inoculated into BALB/c mice (via s.c. injection) to establish a tumor model as indicated in the Materials and Methods section. Mice bearing tumors were randomly assigned to groups (six mice per group) and treated with vehicle, H89 (10 mg/kg), tetrandrine (25 mg/kg) alone or a combination of H89 and tetrandrine (H89 10 mg/kg and tetrandrine 25 mg/kg) every other day. The tumor volume was measured (the bars represent the means ± SD). **b** Scatter plots display the quantitative tumor weights at the end of the experiment. **c** MDA-MB-231 transplanted tumors were dissected and subjected to a TUNEL assay. Scale bars, 100 μm. **d** The level of the oxidative stress marker MDA was measured in the tumor tissues. **e** Cleaved-PARP, Mcl-1 and LC3 were analyzed by Western blot from tumor tissue lysates. **f** BALB/c mice (*n* = 6) were transplanted with MDA-MB-231 Ctrl or Mcl-1 overexpressing cells and treated with DMSO or H89/tetrandrine (H89 10 mg/kg and tetrandrine 25 mg/kg) every other day; tumor volumes were measured during the study. **g** Scatter plots display the quantitative tumor weights. **h** Western blot of tumor lysates from MDA-MB-231 Ctrl or Mcl-1-bearing mice with the indicated treatments. **i** BALB/c mice (*n* = 6) were transplanted with MDA-MB-231 shCtrl or shc-Myc cells and treated as previously described, and the tumor volume and **j** tumor weight are shown. **k** Western blot of tumor lysates from MDA-MB-231 shCtrl or shc-Myc-bearing mice with the indicated treatments. Data are reported as the mean ± SD and were analyzed by Student’s t-test; *n* = 6 mice per group; *P < 0.05, **P < 0.01 and NS = not significant. All images are representative of six mice per group

To assess the therapeutic efficacy of H89 and tetrandrine combination therapy in vivo, we established MDA-MB-231 subcutaneous tumor xenograft models with female athymic nude mice. After six days, tumor-bearing mice were randomly assigned to four groups and were administered vehicle, H89 (10 mg·kg^− 1^), tetrandrine (25 mg·kg^− 1^) or the combination treatment every other day for 28 days. At 18 days, treatment with H89 or tetrandrine inhibited the growth of tumor xenografts; however, there was an enhanced effect in the combination group (Fig. 7a). Consistent with the tumor volumes, the mean tumor weights substantially decreased in the H89/tetrandrine group compared with the other groups (Fig. 7b). Notably, the body weight measurement indicated that the dose of H89/tetrandrine combination was tolerable to the animals (Additional file 1: Figure S4A). Furthermore, TUNEL assays showed significant apoptosis in the tumor tissue from the animals treated with H89/tetrandrine (Fig. 7c). The level of lipid peroxidation product MDA, which indicates the level of oxidative stress in tissues, was increased in the H89/tetrandrine group (Fig. 7d). Consistent with the observations in vitro, H89/tetrandrine increased the levels of cleaved PARP and LC3-II and decreased the levels of Mcl-1 in vivo (Fig. 7e). Next, to examine the role of Mcl-1 in H89/tetrandrine combination treatment in vivo, we performed xenograft assays in MDA-MB-231 ectopically expressing Mcl-1. The tumor regression in response to H89/tetrandrine was statistically significant in the control tumors, but not in the Mcl-1 overexpressing tumors (Fig. 7f and g). Tumor lysates showed the Mcl-1 overexpression group exhibited decreased cleavage of PARP in response to H89/tetrandrine treatment (Fig. 7h). Again, the combination therapy was well tolerated by evidence of weight sustainability (Additional file 1: Figure S4B). To be consistent with the previous experiment, we continued to experiment with MDA-MB-231 and established xenograft models using c-Myc knockdown MDA-MB-231 cells. The in vivo results showed that c-Myc depletion exhibited resistance to H89/tetrandrine treatment (Fig. 7i and j). Consistent with the in vitro findings, c-Myc knockdown increased the Mcl-1 expression in vivo (Fig. 7k). The combination-treated mice did not exhibit a reduction in weight gain during the treatment period (Additional file 1: Figure S4C).

## Discussion

Apoptosis, defined as type-I programmed cell death (PDC), is considered to be a major route by which chemotherapeutic agents eradicate cancer cells [45, 46]. In this study, we showed that H89/tetrandrine activated caspase-dependent apoptosis through mitochondrial-mediated pathways, upregulated the expression of the pro-apoptotic proteins Bim and Bid, and downregulated the expression of the anti-apoptotic protein Mcl-1. Surprisingly, H89/tetrandrine-induced cell death could not be completely reversed by the apoptosis inhibitor z-VAD-fmk, which implies that apoptosis was not the only contributor. Autophagy is involved in type-II programmed cell death, particularly in apoptosis-deficient cells, and may be exploited to suppress tumor growth [47, 48]. Our results showed that H89/tetrandrine-induced cell death was moderately diminished by the autophagy inhibitor, which demonstrates the contribution of autophagy to cell death in response to treatment. Therefore, a combination of agents that induce both apoptotic and autophagic cell death may have greater advantages during the treatment of cancer.

H89 is a strong PKA inhibitor. Moreover, our study suggests that PKA and ERK signaling are involved in the response to the PKA kinase inhibitor H89 and tetrandrine synergistic anti-tumor activity. H89/tetrandrine resulted in almost complete abrogation of the expression of phosphorylated CREB1. Moreover, pretreatment with the adenylyl cyclase activator FSK partially rescued cells from death, which suggests that combined treatment exerted anti-tumor effects in a cAMP/PKA-dependent manner. To mimic the H89-mediated inhibition of PKA, PKI (14–22) amide, another PKA special inhibitor, was used in combination with tetrandrine to treat AGS, 769-P and 786-O cells (Additional file 1: Figure S2D). PKI and tetrandrine also acted synergistically on cancer cells, which implies that the suppression of PKA activity plays a role in the anti-tumor activity of H89 plus tetrandrine.

However, the relationship between PKA and ERK signaling was not investigated in this study. Previous reports have demonstrated that cAMP increases inhibitory Raf-1 phosphorylation at Ser-259 and reduces activating Raf-1 phosphorylation at Ser-338 in a PKA-dependent manner, thereby inducing ERK deactivation [49, 50], which is consistent with our finding that H89 induced PKA inhibition and tetrandrine induced ERK activation are concomitantly involved in H89/tetrandrine combination treatment induced cell death.

Mcl-1 is a pro-survival member of the Bcl-2 family and is highly expressed in various types of malignancy. Thus, Mcl-1 has emerged as a promising target for cancer treatment [51]. In our study, we determined that H89/tetrandrine treatment synergistically inhibited Mcl-1 in cancer cells at both the transcription and protein expression levels. Furthermore, we identified that Mcl-1 plays an important role in H89/tetrandrine anti-tumor activity in vitro and in vivo. Mechanistically, Mcl-1 appears to inhibit apoptosis by preventing mitochondrial dysfunction, with a limited effect on autophagy. However, it is not clear why the expression of the anti-apoptotic protein Mcl-1 was decreased in response to H89/tetrandrine treatment. This finding must be investigated in our future studies.

c-Myc, a commonly activated oncogene, also increases cellular susceptibility to apoptosis [52]. In this study, we interestingly determined that c-Myc-overexpressing cancer cells are more sensitive to H89/tetrandrine combination therapy. Consistently, the knockdown of c-Myc attenuated the sensitivity to H89/tetrandrine in vitro and in mouse xenograft models. We showed that the knockdown of c-Myc significantly increased the Mcl-1 expression, and the overexpression of c-Myc decreased the Mcl-1 levels, which indicates that c-Myc regulating sensitivity to H89/tetrandrine may be associated with downregulating Mcl-1. Other researchers have previously reported that oncogenes such that c-Myc activation induced DNA damage in human normal fibroblasts, which was correlated with the induction of ROS without induction of apoptosis [53]. Having uncovered that c-Myc regulates ROS generation in cancer cells and affects chemotherapeutic sensitivity, in this study, we determined that c-Myc knockdown or ectopic expression significantly diminished or increased ROS generation, respectively. These findings may explain why c-Myc amplified cells are more sensitive to H89/tetrandrine treatment. Although intracellular ROS were increased in AGS and MDA-MB-231 shc-Myc cells when treated with H89/tetrandrine, the levels remained lower than in shCtrl cells, which implies that an appropriate ROS threshold is necessary for H89/tetrandrine induced cell death.

## Conclusions

**Fig. 8:**
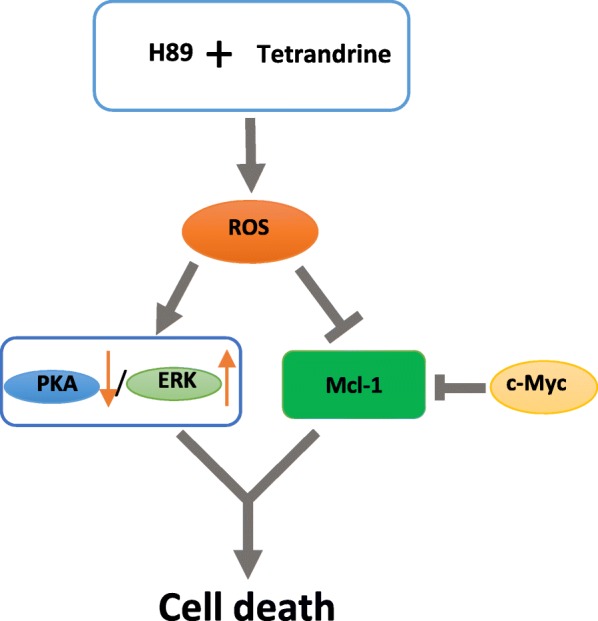
Proposed model of the H89/tetrandrine combination in cancer

Our data indicate that H89/tetrandrine showed a synergistic anti-tumor activity by inducing concomitant cell apoptosis and autophagy in vitro and in vivo. The potential molecular mechanisms involved ROS regulated PKA and ERK signaling and the anti-apoptotic protein Mcl-1 (Fig. 8). Furthermore, c-Myc amplified cells are more sensitive to H89/tetrandrine combined treatment. Thus, the combination of tetrandrine and H89 may be a promising therapeutic strategy for cancer patients and provides a significant clinical application of tetrandrine in the treatment of human cancer. Moreover, this combination provides novel, selectively targeted, therapeutic strategies for patients with c-Myc amplification.

## Additional file

Additional file 1: Supplementary figures and figure legends. (DOCX 1246 kb)

## Abbreviations

3-MA: 3-methyladenine
DCFH-DA: 2′,7′-dichlorodihydrofluorescein diacetate
FSK: Forskolin
NAC: N-acetyl-L-cysteine
PKA: Protein kinase A
PKI: PKI (14–22) amide (myristoylated)
ROS: Reactive oxygen species
Tet: Tetrandrine
