# Supplementary material for: Combination therapy with protein kinase inhibitor H89 and Tetrandrine elicits enhanced synergistic antitumor efficacy
Source: J Exp Clin Cancer Res. 2018 Jun 4;37:114. doi: 10.1186/s13046-018-0779-2 (PMC5987653; doi:10.1186/s13046-018-0779-2)
Supplement: Supplementary file 1 — Supplementary figures and figure legends. (DOCX 1246 kb) [file 13046_2018_779_MOESM1_ESM.docx]

**Supplementary Figures and Figure legends**

**
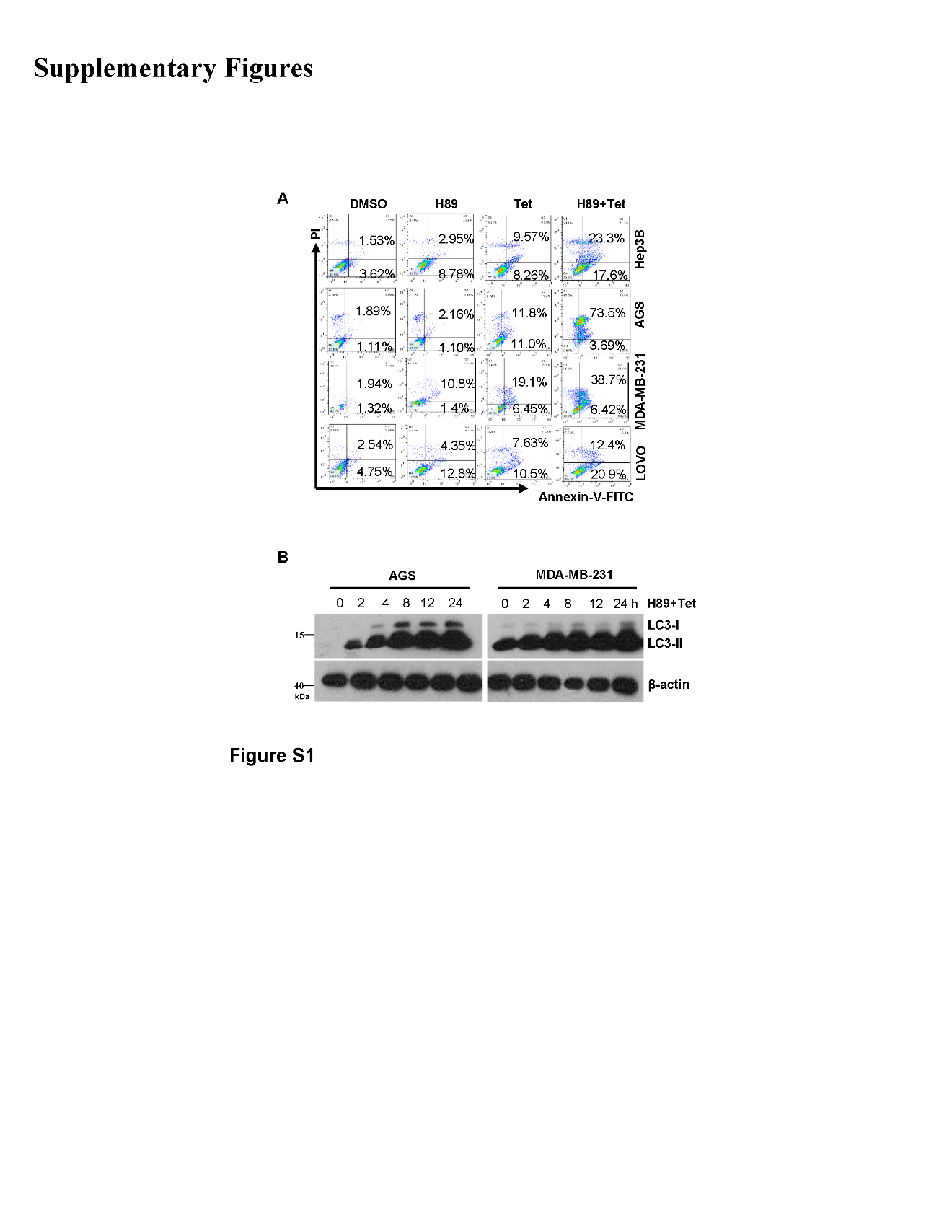
**

**Figure S1** **H89 in combination with tetrandrine synergistically induced concomitant apoptosis and autophagy.** (A) Flow cytometry analysis of apoptosis following treatment with tetrandrine (4 μM) and H89 (6 μM) alone or combination for 48 h in Hep3B, AGS, MDA-MB-231, LOVO. (B) Western blot analysis of LC3 expression following H89/tetrandrine combined treatment in the indicate time. Data are reported as at least three times independent experiments.


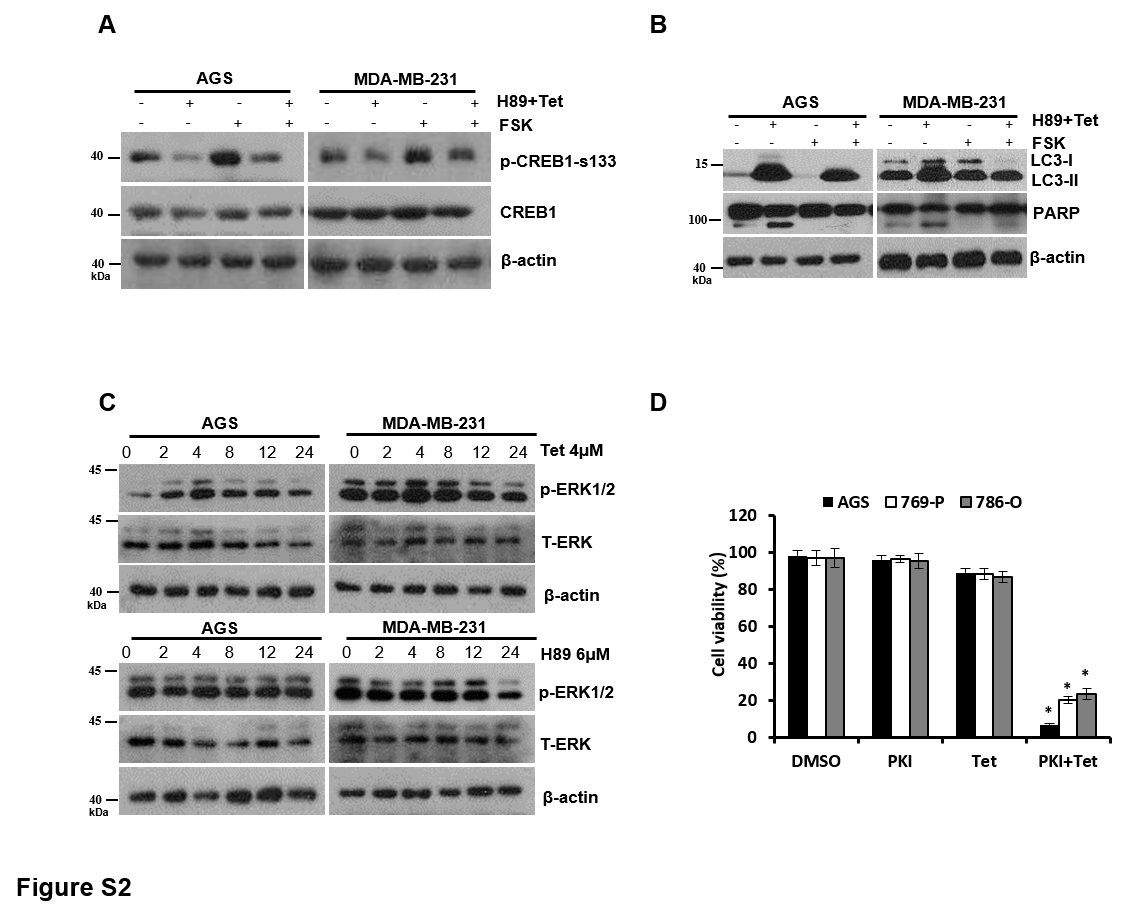


**Figure S2** **PKA and ERK signaling are involved in H89/tetrandrine induced cell death.** (A) AGS and MDA-MB-231 cells were pre-treated with 10 μM FSK for 1 hour followed by combination treatment for 24 h, CREB1 and p-CREB1-s133 were analyzed by Western blot analysis. (B) Analysis of LC3 and PARP levels in AGS and MDA-MB-231 cells pretreated with FSK. (C) The cells were treated with H89 or tetrandrine for (0–24) h. p-ERK, T-ERK were analyzed by Western blot. (D) AGS, 769-P, 786-O cells were incubated with 5 μM PKI and 4 μM tetrandrine or PKI/tetrandrine in combination for 72 h. Cell viabilities were determined. Data are reported as the mean ± SD and analyzed by Student’s t-test; all data were represented at least *n* = 3 independent experiments.

**
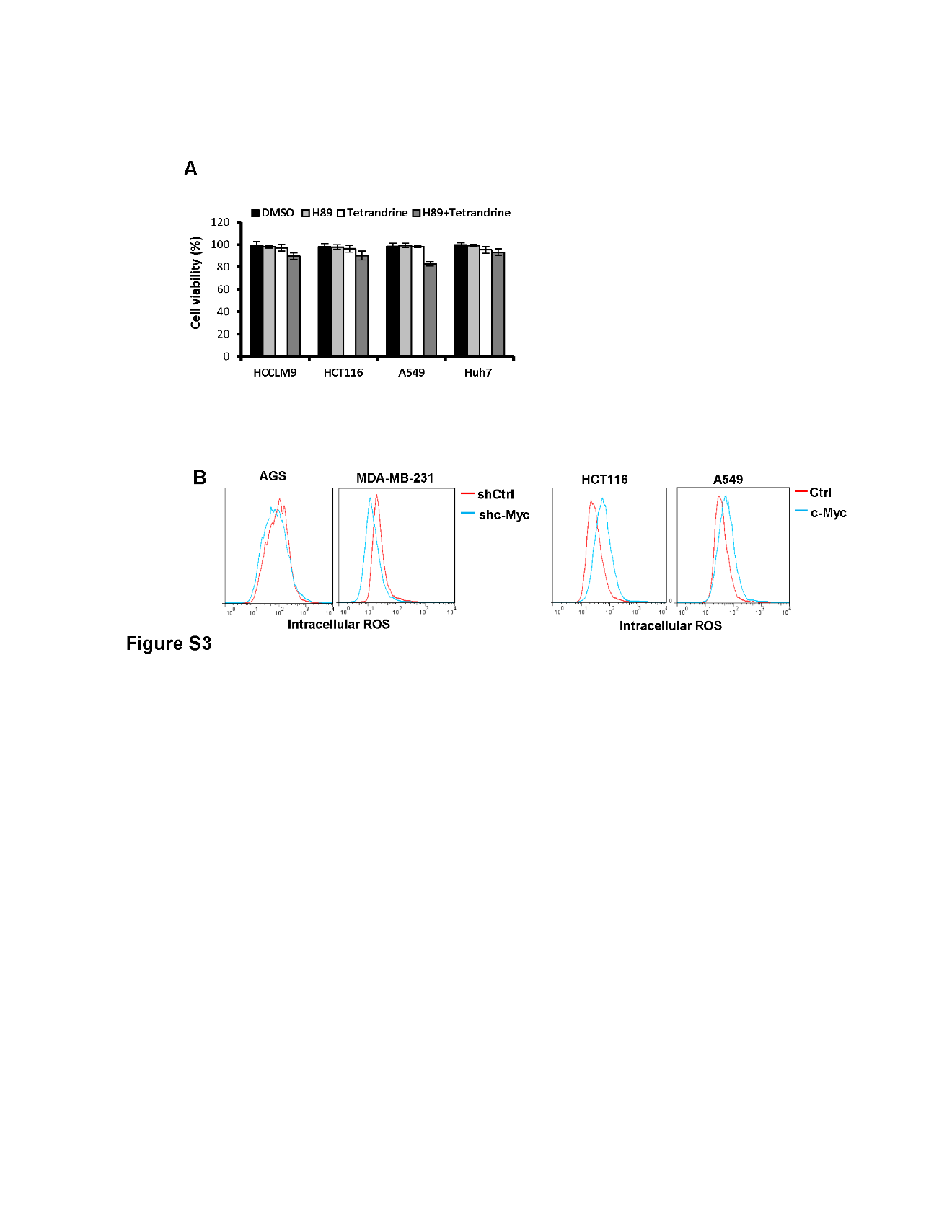
**

**Figure S3** **c-Myc sensitizes cancer cells to H89/tetrandrine combination treatment.** (A) HCCLM9, HCT116, A549 and Huh7 cell lines were treated with H89 or/and for 72 h, followed by assessment for cell viability. (B) AGS, MDA-MB-231 cells transduced with c-Myc shRNA#1 or shCtrl and HCT116, A549 cells engineered to overexpress c-Myc were subsequently treated with control or H89/tetrandrine for 24 h. Intracellular ROS were determined by flow cytometry. Data are reported as the mean ± SD and analyzed by Student’s t-test; all data were represented at least *n* = 3 independent experiments.

**
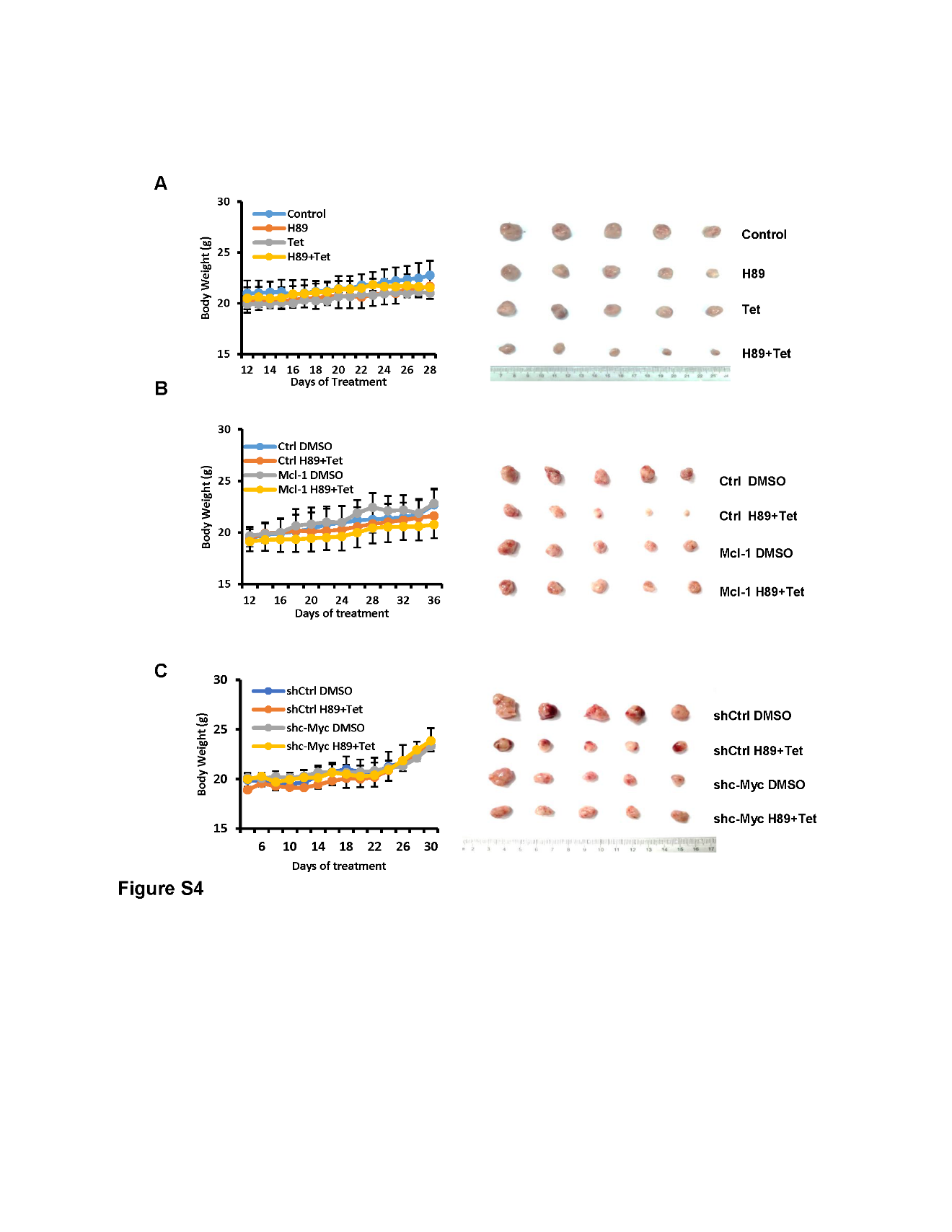
**

**Figure S4 Combination treatment with H89 and tetrandrine leads to a regression of xenograft tumors.** (A) Weights of MDA-MB-231-bearing mice and representative images of xenograft tumors (*n* = 6). (B) Weights of MDA-MB-231 Ctrl or Mcl-1 overexpression-bearing mice and representative tumor images (*n* = 6). (C) Weights of MDA-MB-231 shCtrl or shc-Myc-bearing mice and representative tumor images (*n* = 6). Data are reported as the mean ± SD and analyzed by Student’s t-test.
